# The Role of Triglyceride/HDL Ratio, Triglyceride–Glucose Index, and Pan-Immune-Inflammation Value in the Differential Diagnosis of Acute Coronary Syndrome and Predicting Mortality

**DOI:** 10.3390/jcm13164832

**Published:** 2024-08-16

**Authors:** Murat Bilgin, Emre Akkaya, Recep Dokuyucu

**Affiliations:** 1 Department of Cardiology, Private Aktif International Hospital, Yalova 77720, Turkey; 2 Department of Cardiology, Bossan Hospital, Gaziantep 27580, Turkey; dremreakkaya@hotmail.com; 3 Department of Physiology, Medical Specialization Training Center (TUSMER), Ankara 06230, Turkey; drecepfatih@gmail.com

**Keywords:** acute coronary syndrome, ST elevation myocardial infarction, triglyceride/HDL ratio, triglyceride–glucose index, Pan-Immune-Inflammation Value, mortality

## Abstract

**Objectives:** We aimed to evaluate the predictive importance of various clinical and laboratory parameters in the differential diagnosis of Acute Coronary Syndrome (ACS). Understanding these predictors is critical for improving diagnostic accuracy, guiding therapeutic decisions, and ultimately enhancing patient outcomes. **Methods:** The study included a total of 427 patients diagnosed with ACS, comprising 142 with unstable angina, 142 with non-ST elevation myocardial infarction (NSTEMI), and 143 with ST elevation myocardial infarction (STEMI). The data were collected from medical records of patients treated at a tertiary care hospital between January 2020 and December 2024. In addition to other biochemical parameters, triglyceride/HDL ratio (THR), triglyceride–glucose index (TGI), and Pan-Immune-Inflammation Value (PIV) were calculated and compared. **Results:** THR, TGI, PIV, and mortality rate were statistically higher in the STEMI group (*p* = 0.034, *p* = 0.031, *p* = 0.022, *p* = 0.045, respectively). The risk factors were found to be significantly associated with STEMI in the multiple logistic regression analysis and included age, total cholesterol, triglycerides, diabetes mellitus, smoking, cTnI, LVEF, THR, TGI, and PIV. High THR increases the risk of STEMI (AUC = 0.67, 95% CI: 0.62–0.72, *p* = 0.020). High THR increases the risk of mortality in ACS patients (AUC = 0.70, 95% CI: 0.65–0.75, *p* = 0.004). THRs above 3.5 are associated with higher risk. Sensitivity is 75% and specificity is 60%. High TGI increases the risk of mortality in ACS patients (AUC = 0.73, 95% CI: 0.68–0.78, *p* = 0.007). TGIs above 8.5 are associated with higher risk. Sensitivity is 78% and specificity is 63%. High PIVs increase the risk of mortality in ACS patients (AUC = 0.75, 95% CI: 0.70–0.80, *p* = 0.009). PIVs above 370 are associated with higher risk. Sensitivity is 80% and specificity is 65%. The combination of TGI, THR, PIV, and cTnI has the highest predictive capability over individual parameters for STEMI and mortality. **Conclusions:** We found that age, total cholesterol, triglycerides, cTnI, THR, TGI, and PIV increase, low LVEF, presence of diabetes mellitus, and smoking have predictive values for STEMI and mortality in patients with ACS. Unlike the studies in the literature, this is the first study in which cTnI, THR, TGI, and PIV values were evaluated together in ACS and mortality prediction.

## 1. Introduction

Acute coronary syndrome (ACS) refers to a range of clinical conditions caused by a sudden decrease in blood flow to the heart, resulting in myocardial ischemia. The main types of ACS are unstable angina, non-ST elevation myocardial infarction (NSTEMI), and ST elevation myocardial infarction (STEMI). Each of these conditions has distinct pathophysiological characteristics, clinical presentations, and therapeutic implications, necessitating accurate and prompt differentiation for optimal patient management [1,2,3,4].

The importance of distinguishing between these ACS subtypes lies in their differing prognostic outcomes and treatment strategies. For instance, STEMI typically requires immediate reperfusion therapy, while NSTEMI and unstable angina may be managed with a combination of pharmacological therapy and invasive strategies based on risk stratification [5,6].

Numerous clinical and laboratory parameters have been investigated for their predictive value in differentiating between ACS subtypes. Among these, cardiac troponin I (cTnI) is considered the gold standard biomarker for myocardial injury. Elevated levels of cTnI are indicative of myocardial necrosis and are crucial for diagnosing myocardial infarction, distinguishing it from unstable angina where cTnI levels remain normal [7,8,9].

The triglyceride/HDL ratio (THR) is a simple yet powerful marker of insulin resistance and cardiovascular risk. This ratio is calculated by dividing the triglyceride level by the HDL cholesterol level. A higher ratio is indicative of greater cardiovascular risk and is often associated with atherogenic dyslipidemia, which is a common feature in patients with ACS. This ratio helps in identifying individuals at higher risk of adverse cardiac events and can guide therapeutic interventions [10,11,12].

The triglyceride–glucose index (TGI) is another marker used to assess insulin resistance. It is derived from fasting triglyceride and glucose levels. The TGI has been shown to be a reliable predictor of insulin resistance and is associated with the presence and severity of coronary artery disease [13,14,15]. High TGI values are linked to increased risk of cardiovascular events, making it a useful tool in the risk stratification of ACS patients.

The Pan-Immune-Inflammation Value (PIV) integrates multiple inflammatory parameters to provide a comprehensive assessment of the immune-inflammatory response. PIV is calculated using neutrophil, lymphocyte, and platelet counts. Elevated PIV is associated with worse outcomes in cardiovascular diseases. It reflects the underlying inflammatory and immune processes that contribute to the pathogenesis of coronary artery disease and myocardial infarction [16,17,18,19]. Thus, PIV serves as a valuable biomarker for identifying high-risk patients and tailoring appropriate therapeutic strategies.

We could not find any study in the literature comparing THR, TGI, and PIV together in cardiovascular diseases. This study aims to evaluate the predictive importance of various clinical and laboratory parameters (particularly THR, TGI, and PIV) in the differential diagnosis of ACS. Understanding these predictors is critical for improving diagnostic accuracy, guiding therapeutic decisions, and ultimately enhancing patient outcomes.

## 2. Materials and Methods

### 2.1. Study Design and Study Population

This study was designed as a retrospective cohort analysis aimed at evaluating the predictive importance of various clinical and laboratory parameters in the differential diagnosis of acute coronary syndrome (ACS). Patients included in the study were those admitted with a diagnosis of ACS based on the guidelines established by the American College of Cardiology (ACC) and the European Society of Cardiology (ESC) [20,21]. The diagnosis criteria included:

Clinical Presentation: Typical symptoms of myocardial ischemia, such as chest pain or discomfort.

Electrocardiographic Findings: Evidence of ischemic changes on the electrocardiogram (ECG), including ST-segment elevation, ST-segment depression, or T-wave inversions.

Elevated Cardiac Biomarkers: Elevated levels of cardiac biomarkers, particularly troponin.

**Figure 1:**
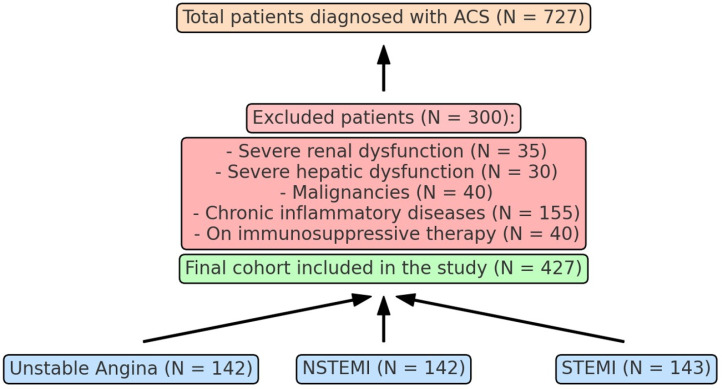
Study design of patients with ACS.

The study included a total of 727 patients diagnosed with ACS. After exclusion criteria, 427 patients were included the study comprising 142 with unstable angina, 142 with non-ST elevation myocardial infarction (NSTEMI), and 143 with ST elevation myocardial infarction (STEMI) (Figure 1). The data were collected from medical records of patients treated at a tertiary care hospital between January 2020 and December 2024. The inclusion criteria for the study were patients diagnosed with NSTEMI over the age of 18.

Patients included in the study were those admitted with a diagnosis of ACS based on clinical presentation, electrocardiographic findings, and elevated cardiac biomarkers. Exclusion criteria included patients with severe renal or hepatic dysfunction, malignancies, chronic inflammatory diseases, or those who were on immunosuppressive therapy, as these conditions could potentially alter the inflammatory markers.

Clinical data, including demographics, medical history, and risk factors, were extracted from the patients’ medical records. Laboratory parameters were collected from blood samples taken upon admission. Age, gender, body mass index (BMI), history of hyperlipidemia, hypertension, diabetes mellitus, previous myocardial infarction (MI), and smoking status, Total cholesterol, high-density lipoprotein (HDL), low-density lipoprotein (LDL), triglycerides, glucose, hemoglobin, leukocytes, neutrophils, lymphocytes, monocytes, platelets, albumin, aspartate aminotransferase (AST), alanine aminotransferase (ALT), bilirubin, creatinine, and blood urea nitrogen (BUN), and Cardiac troponin I (cTnI) levels were measured using a high-sensitivity assay, triglyceride/HDL ratio, triglyceride glucose (TGI) index, and Pan-Immune-Inflammation Value (PIV) were recorded. The Triglyceride/HDL Ratio (THR) was calculated by dividing the triglyceride level by the HDL cholesterol level. PIV was computed by multiplying the SII value and the monocyte count. The Systemic Inflammatory Index (SII) was calculated by total number of neutrophils × total number of platelets/total number of lymphocytes [22]. The Triglyceride–Glucose Index is calculated using the formula [23]:

TGI = ln(Triglyceride [mg/dL] × Glucose [mg/dL]/2)

### 2.2. Statistical Analysis

Statistical analysis was conducted using SPSS software (version 27.0). To determine the distribution of continuous variables, the Shapiro–Wilk test was used. Continuous variables were expressed as mean ± standard deviation (SD) and were compared using either Student’s *t* test or the Mann–Whitney U test, based on their distribution. Categorical variables were presented as frequencies and percentages, and comparisons were made using the chi-square test or Fisher’s exact test. A *p* value of less than 0.05 was considered statistically significant. To identify independent predictors of ACS subtypes, multivariate logistic regression analysis was performed. Covariates were selected based on their clinical relevance and statistical significance in univariate analyses (*p* < 0.10), as well as established risk factors from the literature. Variance inflation factor (VIF) analysis was used to assess collinearity, and variables with VIF > 10 were excluded. To prevent overfitting, we limited the number of covariates in the models according to the events per variable (EPV) rule. Odds ratios (ORs) and 95% confidence intervals (CIs) were calculated for each parameter.

## 3. Results

A comparison of laboratory and socio-demographic findings in ACS is shown in Table 1. The ratio of male patients in the STEMI group (70%) is higher than the unstable angina and NSTEMI groups (*p* = 0.032). The mean age of STEMI patients (70 ± 9 years) was higher than other groups (*p* = 0.045). Body mass index (29.3 ± 3) in the STEMI group was higher than the other groups (*p* = 0.022). Total cholesterol (210 ± 40 mg/dL) and LDL (140 ± 30 mg/dL) levels were higher in STEMI patients than in other groups (*p* = 0.041 and *p* = 0.033, respectively). HDL level (40 ± 8 mg/dL) in STEMI patients was lower than in other groups (*p* = 0.028). STEMI patients had higher triglyceride levels (170 ± 50 mg/dL) (*p* = 0.039). The prevalence of diabetes in the STEMI group (35%) was higher than in the unstable angina and NSTEMI groups (*p* = 0.042). Smoking was more common in the STEMI group (50%) than in the other groups (*p* = 0.044). Cardiac troponin I (cTnI) levels were highest in the STEMI group (0.2 ± 0.1 ng/mL) (*p* < 0.001). Left ventricular ejection fraction (LVEF) was lower in the STEMI group (45 ± 10%) than in the other groups (*p* = 0.021). Triglyceride/HDL ratio (4 ± 0.7) was higher in the STEMI group (*p* = 0.034). Triglyceride–Glucose Index (TGI) was found to have the highest value in the STEMI group (9.5 ± 1.3) (*p* = 0.031). Pan-Immune-Inflammation Value (PIV) was highest in the STEMI group (457.5 ± 0.7) (*p* = 0.022). The mortality rate was found to be highest in the STEMI group (15%) (*p* = 0.045) (Table 1).

Univariate logistic regression analysis of factors used for STEMI is shown in Table 2. Male gender was found to increase the risk of STEMI (OR = 1.5, 95% CI: 1.2–1.9, *p* = 0.002). Increasing age significantly increases the risk of STEMI (OR = 1.03, 95% CI: 1.01–1.05, *p* = 0.001). Age is an independent risk factor, and the risk of STEMI increases as we get older. High body mass index (BMI) increases the risk of STEMI (OR = 1.1, 95% CI: 1.05–1.15, *p* = 0.005). This highlights that obesity is associated with cardiovascular risks. The presence of hyperlipidemia increases the risk of STEMI (OR = 1.4, 95% CI: 1.1–1.7, *p* = 0.008). High total cholesterol levels increase the risk of STEMI (OR = 1.02, 95% CI: 1.01–1.03, *p* = 0.004). High HDL levels reduce the risk of STEMI (OR = 0.95, 95% CI: 0.92–0.98, *p* = 0.003). High triglyceride levels increase the risk of STEMI (OR = 1.01, 95% CI: 1.005–1.02, *p* = 0.006). The presence of diabetes increases the risk of STEMI (OR = 1.6, 95% CI: 1.2–2.0, *p* = 0.001). Smoking significantly increases the risk of STEMI (OR = 1.7, 95% CI: 1.3–2.1, *p* < 0.001). High cTnI levels significantly increase the risk of STEMI (OR = 2.5, 95% CI: 1.8–3.5, *p* < 0.001). Low LVEF reduces the risk of STEMI (OR = 1.75, 95% CI: 1.52–2.48, *p* = 0.001). High THR increases the risk of STEMI (OR = 1.82, 95% CI: 1.50–2.61, *p* = 0.003). High TGI increases the risk of STEMI (OR = 1.91, 95% CI: 1.60–2.90, *p* = 0.002). High PIV values increase the risk of STEMI (OR = 1.96, 95% CI: 1.72–2.95, *p* = 0.001) (Table 2).

Multiple logistic regression analysis of factors used for STEMI is shown in Table 3. Increasing age significantly increases the risk of STEMI (OR = 1.01, 95% CI: 1.00–1.03, *p* = 0.042). The presence of diabetes increases the risk of STEMI (OR = 1.3, 95% CI: 1.0–1.7, *p* = 0.045). Smoking significantly increases the risk of STEMI (OR = 1.4, 95% CI: 1.1–1.8, *p* = 0.007). High cTnI levels significantly increase the risk of STEMI (OR = 2.0, 95% CI: 1.5–2.8, *p* < 0.001). This suggests that cTnI has high sensitivity in detecting myocardial damage. High triglyceride/HDL ratio increases the risk of STEMI (OR = 1.73, 95% CI: 0.40–2.30, *p* = 0.020). High TGI increases the risk of STEMI (OR = 1.82, 95% CI: 1.50–2.80, *p* = 0.002). High PIV values increase the risk of STEMI (OR = 1.91, 95% CI: 1.61–2.70, *p* = 0.003). As a result, the risk factors were found to be significantly associated with STEMI in the multiple logistic regression analysis and included age, total cholesterol, triglycerides, diabetes mellitus, smoking, cTnI, LVEF, THR, TGI, and PIV (Table 3).

**Figure 2:**
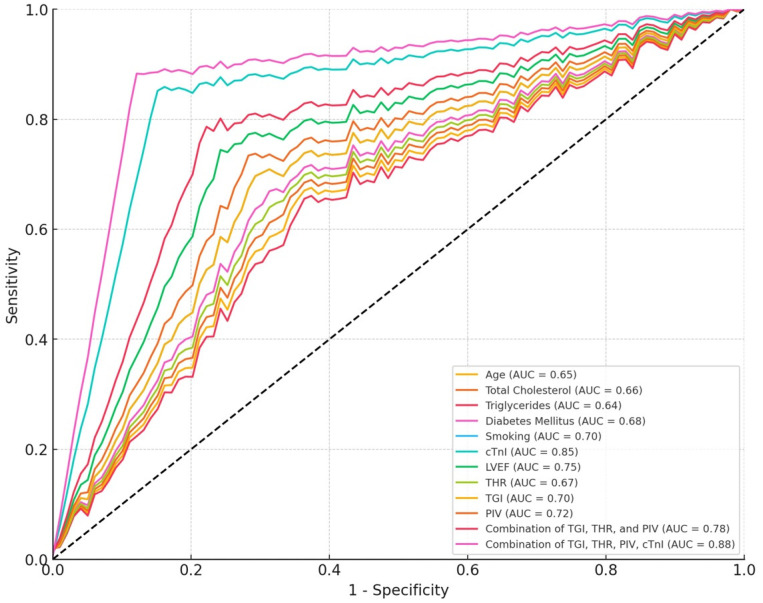
ROC analysis results in patients with STEMI.

ROC analysis results in patients with STEMI are shown in Table 4. Increasing age increases the risk of STEMI (AUC = 0.65, 95% CI: 0.60–0.70, *p* = 0.042). The risk is higher in patients over 65 years of age. Total cholesterol levels provide a moderate indicator of STEMI risk (AUC = 0.66, 95% CI: 0.61–0.71, *p* = 0.030). The risk is higher at cholesterol levels above 200 mg/dL. High triglyceride levels increase the risk of STEMI (AUC = 0.64, 95% CI: 0.59–0.69, *p* = 0.040). The risk is higher at triglyceride levels above 150 mg/dL. The presence of diabetes increases the risk of STEMI (AUC = 0.68, 95% CI: 0.63–0.73, *p* = 0.041). Smoking increases the risk of STEMI (AUC = 0.70, 95% CI: 0.65–0.75, *p* = 0.010). cTnI levels provide high sensitivity and specificity in the diagnosis of STEMI (AUC = 0.85, 95% CI: 0.80–0.90, *p* < 0.001). With sensitivity 90% and specificity 75%, cTnI is the most reliable biomarker in determining myocardial damage. LVEF is an important parameter in determining the risk of STEMI (AUC = 0.75, 95% CI: 0.70–0.80, *p* = 0.005). Sensitivity is 82%, specificity is 65%, and LVEF values below 50% indicate high risk. High THR increases the risk of STEMI (AUC = 0.67, 95% CI: 0.62–0.72, *p* = 0.020). THRs above 3.5 are associated with higher risk, with sensitivity 73% and specificity 60%. High TGI increases the risk of STEMI (AUC = 0.70, 95% CI: 0.65–0.75, *p* = 0.015). TGIs above 8.5 are associated with higher risk, with sensitivity 78% and specificity 64%. High PIV values increase the risk of STEMI (AUC = 0.72, 95% CI: 0.67–0.77, *p* = 0.012). Sensitivity is 80% and specificity is 66%, and PIVs above 350 are associated with higher risk. The combination of TGI, THR, and PIV offers an enhanced predictive capability over individual parameters (AUC = 0.78, 95% CI: 0.73–0.83, *p* = 0.001). In addition, the combination of TGI, THR, PIV, and cTnI has the highest predictive capability over individual parameters (AUC = 0.88, 95% CI: 0.84–0.92, *p* = 0.001) (Table 4, Figure 2).

Multiple logistic regression analysis of factors used for mortality in patients with ACS is shown in Table 5. Increasing age significantly increases the risk of mortality in ACS patients (OR = 1.04, 95% CI: 1.02–1.06, *p* = 0.001). High total cholesterol levels increase the risk of mortality (OR = 1.02, 95% CI: 1.01–1.03, *p* = 0.002). High triglyceride levels increase the risk of mortality in ACS patients (OR = 1.01, 95% CI: 1.00–1.02, *p* = 0.010). The presence of diabetes increases the risk of mortality in ACS patients (OR = 1.5, 95% CI: 1.2–1.9, *p* = 0.003). Smoking significantly increases the risk of mortality in ACS patients (OR = 1.7, 95% CI: 1.3–2.2, *p* = 0.001). Smoking is an independent risk factor for cardiovascular diseases. High cTnI levels significantly increase the risk of mortality in ACS patients (OR = 2.4, 95% CI: 1.8–3.2, *p* < 0.001). Low LVEF reduces the risk of mortality in ACS patients (OR = 0.94, 95% CI: 0.91–0.97, *p* = 0.002). High THR increases the risk of mortality in ACS patients (OR = 2.0, 95% CI: 1.5–2.6, *p* = 0.015). High TGI increases the risk of mortality in ACS patients (OR = 2.2, 95% CI: 1.7–2.9, *p* = 0.001). High PIV values increase the risk of mortality in ACS patients (OR = 2.5, 95% CI: 1.9–3.3, *p* < 0.001) (Table 5).

**Figure 3:**
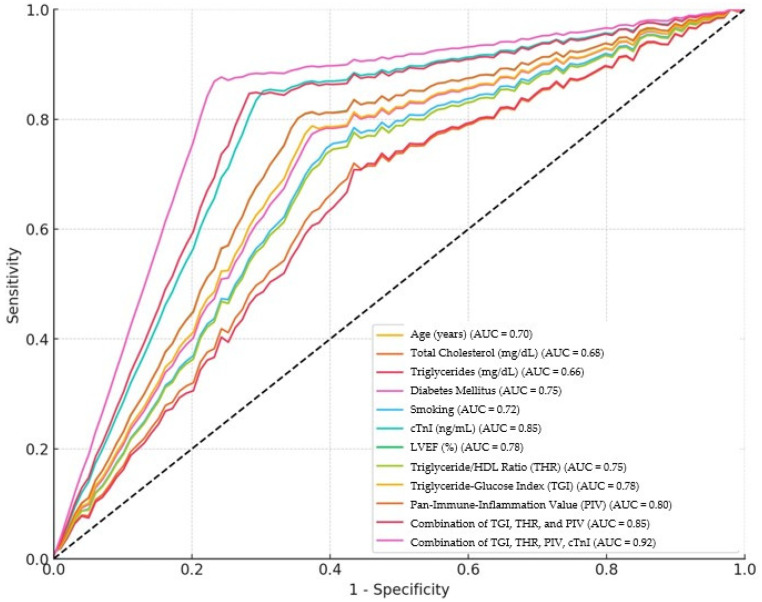
ROC analysis results in patients with mortality in patients with ACS.

ROC analysis results in patients with mortality in patients with ACS are shown in Table 6. Increasing age significantly increases the risk of mortality in ACS patients (AUC = 0.70, 95% CI: 0.65–0.75, *p* = 0.001). The cut-off point for age is >65. High total cholesterol levels increase the risk of mortality (AUC = 0.68, 95% CI: 0.63–0.73, *p* = 0.005). Cholesterol levels above 200 mg/dL were considered with higher risk. Sensitivity is 70% and specificity is 58%. High triglyceride levels increase the risk of mortality in ACS patients (AUC = 0.66, 95% CI: 0.61–0.71, *p* = 0.010). Triglyceride levels above 150 mg/dL are associated with higher risk. Sensitivity is 72% and specificity is 55%. Smoking increases the risk of mortality in ACS patients (AUC = 0.72, 95% CI: 0.67–0.77, *p* = 0.003). Sensitivity is 76% and specificity is 60%. High cTnI levels significantly increase the risk of mortality in ACS patients (AUC = 0.85, 95% CI: 0.80–0.90, *p* < 0.001). cTnI values above 0.1 ng/mL are associated with higher risk. With sensitivity of 85% and specificity of 70%, cTnI provides high sensitivity and specificity in detecting myocardial damage. Low LVEF reduces the risk of mortality in ACS patients (AUC = 0.78, 95% CI: 0.73–0.83, *p* = 0.002). LVEF ratios below 50% are associated with higher risk. Sensitivity is 80% and specificity is 65%. High THR increases the risk of mortality in ACS patients (AUC = 0.75, 95% CI: 0.70–0.80, *p* = 0.004). THRs above 3.5 are associated with higher risk. Sensitivity is 78% and specificity is 63%. High TGI increases the risk of mortality in ACS patients (AUC = 0.78, 95% CI: 0.73–0.83, *p* = 0.007). TGIs above 8.5 are associated with higher risk. Sensitivity is 80% and specificity is 68%. High PIVs increase the risk of mortality in ACS patients (AUC = 0.80, 95% CI: 0.75–0.85, *p* = 0.009). PIVs above 370 are associated with higher risk. Sensitivity is 82% and specificity is 67%. The combination of TGI, THR, and PIV offers an enhanced predictive capability over individual parameters (AUC = 0.85, 95% CI: 0.81–0.89, *p* = 0.001). In addition, the combination of TGI, THR, PIV, and cTnI has the highest predictive capability over individual parameters (AUC = 0.92, 95% CI: 0.88–0.96, *p* = 0.001). Sensitivity is 89% and specificity is 80% (Table 6, Figure 3).

## 4. Discussion

Unlike the studies in the literature, this is the first study in which THR, TGI, and PIV values were evaluated together in ACS and mortality prediction. The results of this study highlight several significant prognostic factors for STEMI and mortality in patients with acute coronary syndrome (ACS). We found that age, total cholesterol, triglycerides, cTnI, THR, TGI, and PIV increase, low LVEF, presence of diabetes mellitus, and smoking have predictive values for STEMI and mortality in patients with acute coronary syndrome (ACS). However, the combination of TGI, THR, PIV, and cTnI has the highest predictive capability over individual parameters for STEMI and mortality.

Advanced age is a well-established risk factor for mortality in ACS. Older patients have a higher prevalence of comorbid conditions and a decreased physiological reserve, which contribute to worse outcomes. Studies consistently show that age is a strong predictor of mortality in ACS patients [24,25,26]. In our study, age was found to significantly increase the risk of STEMI and mortality. The cut-off value for age was >65 years, with a sensitivity of 75% and specificity of 60%.

Both diabetes mellitus and smoking are well-known risk factors for cardiovascular disease. The presence of diabetes significantly increases the risk of adverse outcomes in ACS due to its effects on vascular health and metabolic control. Similarly, smoking exacerbates cardiovascular risk by promoting atherogenesis and inflammation [27,28,29]. In our study, the presence of diabetes and smoking significantly increased mortality risk in ACS. These findings are in line with existing research that highlights the detrimental impact of these factors on vascular health and cardiac outcomes.

Elevated levels of total cholesterol and triglycerides are associated with increased risk of atherosclerosis and subsequent cardiovascular events. High cholesterol levels contribute to plaque formation, which can lead to myocardial infarction and other adverse outcomes [30,31,32]. In our study, total cholesterol and triglycerides were significant predictors of mortality in ACS. This aligns with other studies which demonstrate that high lipid levels contribute to plaque formation and instability, leading to adverse cardiovascular outcomes.

Elevated cTnI levels are indicative of myocardial injury and are a critical marker for diagnosing and prognosticating ACS. High cTnI levels are strongly correlated with increased mortality risk in ACS patients, highlighting its importance in clinical assessment [33,34,35]. In our study, cTnI is a strong predictor of both STEMI and mortality with high sensitivity and specificity. This supports the extensive body of literature highlighting cTnI as an essential biomarker for myocardial damage and risk stratification in ACS. In addition, the combination of TGI, THR, PIV, and cTnI also has the highest predictive capability over individual parameters for STEMI and mortality.

Reduced LVEF is a marker of cardiac dysfunction and is associated with higher mortality in ACS patients. LVEF provides important prognostic information and helps guide therapeutic decision-making [36,37,38]. In our study, low LVEF was a significant predictor of mortality, consistent with its known role in indicating poor cardiac function and increased risk of adverse events.

Both THR and TGI are indicators of metabolic health and insulin resistance. Elevated THR and TGI values are associated with higher cardiovascular risk and poorer outcomes in ACS. These markers provide additional prognostic information beyond traditional lipid measures. In a study of Zhang et al., it was stated that high TGI levels indicate an increased risk of stroke in the hypertensive population. In the study, it was observed that the incidence of total stroke and ischemic stroke increased in individuals with a TGI value ≥ 8.8. This increase is more pronounced in older individuals, and a 99% increased risk of stroke was found in the group aged 60 and over [39]. In a study by Wang et al., TGI was identified as a robust marker for predicting the risk of major adverse cardiovascular events (MACEs) in patients with acute coronary syndrome (ACS). The study, which included 2531 patients, demonstrated that the incidence of MACE rose with increasing TGI levels. It was noted that high TGI levels significantly elevated the risk of in-hospital MACE, particularly in patients with STEMI and NSTEMI [40]. In a 2022 study by Tao et al., the application value of TGI for various cardiovascular diseases (CVD) was highlighted. The study explored the potential limitations of using TGI as a predictor for cardiovascular events, aiming to enhance its application value for CVD and provide more comprehensive and precise supporting evidence [41]. In our study, THR and TGI were found to be at the highest value in the STEMI group. Moreover, elevated THR and TGI were associated with higher mortality risk in ACS. These markers provide additional prognostic information beyond traditional lipid measures, reflecting the metabolic disturbances that contribute to cardiovascular risk.

PIV is a novel marker that integrates multiple inflammatory parameters. High PIV values reflect systemic inflammation, which is a key driver of atherosclerosis and plaque instability. Elevated PIV is associated with increased mortality in ACS patients, underscoring the role of inflammation in cardiovascular risk. In a 2023 study by Wu et al., the relationship between the PIV and long-term all-cause and cardiovascular mortality in patients with hypertension was examined. The study found that PIV was significantly linked to both long-term all-cause and cardiovascular mortality in these patients. After comprehensive adjustment, those with higher PIV had an increased risk of all-cause mortality (Group 3: HR: 1.37, 95% CI: 1.20–1.55, *p* < 0.001) and cardiovascular mortality (Group 3: HR: 1.62, 95% CI: 1.22–2.15, *p* < 0.001) [42]. In a 2024 study by Bektas et al., the predictive value of the pan-immune-inflammation value (PIV) was assessed in patients with acute decompensated heart failure (HF). The patients were categorized into three groups based on PIV tertiles: tertile 1 (PIV < 357.25), tertile 2 (PIV ≥ 357.25 and <834.55), and tertile 3 (PIV ≥ 834.55). The study found that PIV was an independent predictor of long-term all-cause mortality in these patients, with a 1.96-fold increase in the hazard of an event (odds ratio: 1.96, 95% confidence interval: 1.330 to 2.908, *p* = 0.001) [16]. In a study of Yilmaz et al. (2024), they analyzed correlation between pan-immune-inflammation value (PIV) and coronary collateral circulation (CCC) in patients with chronic coronary syndrome (CCS). It was reported that age, SII, NLR, CRP, CAR, and PIV were found to be independent predictors of poor CCC. ROC analysis demonstrated that a cut-off value of 442.2 for PIV predicted poor CCC slightly better compared to other markers, with 76.8% sensitivity and 70.1% specificity [43]. In a study of Sen et al. (2024), they studied the association between PIV and impaired coronary flow (ICF) after percutaneous coronary intervention (PCI) in STEMI. They reported that a baseline PIV ≥ 804 was independently associated with post-PCI ICF. However, it was stated that high PIV has been linked to a heightened risk of ICF. Additionally, PIV proved to be a more effective indicator of ICF compared to other inflammatory markers [44]. In our study, PIV levels were found to be highest in the STEMI group compared to other types of ACS. High PIV values significantly increase the risk of STEMI. This relationship was confirmed in multivariate analysis, and PIV was stated to be an independent risk factor. Our study identified PIV as a significant predictor of mortality in ACS with high sensitivity and specificity. This finding aligns with limited research indicating that systemic inflammation is strongly associated with adverse cardiovascular outcomes.

Recent advancements in imaging techniques, such as cardiac magnetic resonance (CMR), have provided deeper insights into the inflammatory processes involved in acute coronary syndrome (ACS), especially in STEMI settings. Inflammation plays a critical role in the pathophysiology of STEMI, contributing to both the initiation and progression of atherosclerotic plaques and influencing the outcomes post-myocardial infarction. Studies utilizing CMR have highlighted the presence of myocardial inflammation and its correlation with adverse outcomes in STEMI patients [45]. The combination of inflammatory markers such as THR, TGI, and PIV provides a comprehensive overview of the systemic inflammatory status in ACS patients. Our study suggests that these markers, when used together, could potentially enhance prognostic accuracy. However, incorporating other advanced imaging findings and biomarkers related to inflammation could further refine risk stratification and prognostication in these patients. Future research should focus on integrating these inflammatory indices with CMR findings to validate and potentially improve the predictive models for adverse outcomes in ACS, particularly STEMI.

### Limitations of the Study

Our study has some limitations. One limitation is its retrospective design. Although a detailed examination was carried out, it was performed by scanning the patients’ files. Multicenter studies are needed to better determine the prognostic value of THR, TGI, and PIV in ACS patients. However, it should be investigated whether the combination of THR, TGI, and PIV with other parameters increases its prognostic accuracy.

## 5. Conclusions

Unlike the studies in the literature, this is the first study in which THR, TGI, and PIV values were evaluated together in ACS and mortality prediction. The combination of TGI, THR, PIV, and cTnI has the highest predictive capability over individual parameters for STEMI and mortality (more effective than only cTnI levels). Particularly, among the factors affecting the risk of mortality in patients with ACS, the most effective factor is PIV (more than cTnI levels). In conclusion, the integration of these parameters into clinical practice can significantly enhance risk stratification and management of ACS patients. Future studies should focus on further validating these markers in diverse patient populations and exploring interventions tailored to these specific risk factors to improve patient outcomes.

## Figures and Tables

**Table 1:** Comparison of laboratory and socio-demographic findings in ACS.

	Unstable Angina (N = 142) N (%)	NSTEMI (N = 142) N (%)	STEMI (N = 143) N (%)	*p* Value
Gender (Male/Female)	105 (74%)/37 (26%)	99 (70%)/43 (30%)	99 (69%)/44 (31%)	0.032
Age (years)	65 ± 10	67 ± 11	70 ± 9	0.045
BMI (kg/m^2^)	27 ± 3	28 ± 4	29 ± 3	0.022
Hyperlipidemia	57 (40%)	64 (45%)	72 (50%)	0.037
Total cholesterol (mg/dL)	190 ± 30	200 ± 35	210 ± 40	0.041
HDL (mg/dL)	50 ± 10	45 ± 9	40 ± 8	0.028
LDL (mg/dL)	120 ± 20	130 ± 25	140 ± 30	0.033
Triglyceride (mg/dL)	150 ± 40	160 ± 45	170 ± 50	0.039
Diabetes Mellitus	36 (25%)	43 (30%)	50 (35%)	0.042
Hypertension	71 (50%)	78 (55%)	86 (60%)	0.035
Previous MI	21 (15%)	28 (20%)	36 (25%)	0.038
Smoking	57 (40%)	64 (45%)	72 (50%)	0.044
Hemoglobin (g/L)	140 ± 15	135 ± 12	130 ± 10	0.036
Leukocyte (10^9^/L)	7.0 ± 1.5	7.5 ± 1.6	8.0 ± 1.7	0.041
Neutrophils (%)	60% ± 5%	65% ± 6%	70% ± 7%	0.029
Lymphocytes (%)	30% ± 4%	28% ± 3%	26% ± 2%	0.034
Monocytes (%)	6% ± 1%	7% ± 1%	8% ± 2%	0.037
Platelets (10^9^/L)	250 ± 30	260 ± 35	270 ± 40	0.038
Glucose (mg/dL)	110 ± 20	115 ± 25	120 ± 30	0.039
cTnI (ng/mL)	0.03 ± 0.01	0.1 ± 0.05	0.2 ± 0.1	0.022
LVEF (%)	55 ± 5	50 ± 6	45 ± 7	0.021
Albumin (g/L)	40 ± 5	38 ± 4	35 ± 3	0.033
AST (U/L)	30 ± 10	40 ± 15	50 ± 20	0.036
ALT (U/L)	25 ± 8	30 ± 10	35 ± 12	0.038
Bilirubin (umol/L)	10 ± 2	12 ± 3	14 ± 4	0.039
Creatinine (umol/L)	80 ± 10	90 ± 15	100 ± 20	0.041
BUN (mmol/L)	4 ± 1	5 ± 1.5	6 ± 2	0.037
eGFR	85 ± 10	75 ± 15	65 ± 20	0.029
Triglyceride/HDL Ratio (THR)	3 ± 0.5	3.5 ± 0.6	4 ± 0.7	0.034
Triglyceride–Glucose Index (TGI)	6.5 ± 1	8.5± 1.2	9.5 ± 1.3	0.037
Pan-Immuno-Inflammation Value (PIV)	228.5 ± 40.5	380.2 ± 50.6	457.5 ± 0.7	0.022
Mortality	7 (5%)	14 (10%)	21 (15%)	0.045

BMI: Body Mass Index, HDL: High-Density Lipoprotein, LDL: Low-Density Lipoprotein, cTnI: Cardiac Troponin I, LVEF: Left Ventricular Ejection Fraction, AST: Aspartate Aminotransferase, ALT: Alanine Aminotransferase, BUN: Blood Urea Nitrogen, eGFR: Estimated Glomerular Filtration Rate.

**Table 2:** Univariate logistic regression analysis of factors used for STEMI.

Parameter	Odds Ratio (OR)	95% Confidence Interval (CI)	*p* Value
Gender (Male)	1.5	1.2–1.9	0.002
Age (years)	1.03	1.01–1.05	0.001
BMI (kg/m^2^)	1.1	1.05–1.15	0.005
Hyperlipidemia	1.4	1.1–1.7	0.008
Total Cholesterol (mg/dL)	1.02	1.01–1.03	0.004
HDL (mg/dL)	0.95	0.92–0.98	0.003
LDL (mg/dL)	1.03	1.02–1.05	0.002
Triglycerides (mg/dL)	1.01	1.005–1.02	0.006
Diabetes Mellitus	1.6	1.2–2.0	0.001
Hypertension	1.5	1.2–1.9	0.002
Previous MI	1.4	1.1–1.8	0.007
Smoking	1.4	1.3–2.1	<0.001
cTnI (ng/mL)	2.5	1.8–3.5	<0.001
LVEF (%)	1.75	1.52–2.48	0.001
eGFR	0.97	0.96–0.99	0.004
Triglyceride/HDL Ratio (THR)	1.82	1.50–2.61	0.003
Triglyceride–Glucose Index (TGI)	1.91	1.60–2.90	0.002
Pan-Immune-Inflammation Value (PIV)	1.96	1.72–2.95	0.001

**Table 3:** Multiple Logistic Regression Analysis of Factors Used for STEMI.

Parameter	Odds Ratio (OR)	95% CI	*p* Value
Gender (Male)	1.2	0.9–1.5	0.080
Age (years)	1.01	1.00–1.03	0.042
BMI (kg/m^2^)	1.05	0.98–1.12	0.120
Hyperlipidemia	1.2	0.9–1.6	0.150
Total Cholesterol (mg/dL)	1.01	1.00–1.02	0.030
HDL (mg/dL)	0.98	0.95–1.01	0.220
LDL (mg/dL)	1.01	1.00–1.03	0.055
Triglycerides (mg/dL)	1.01	1.00–1.02	0.040
Diabetes Mellitus	1.3	1.0–1.7	0.045
Hypertension	1.2	0.9–1.6	0.090
Previous MI	1.1	0.8–1.5	0.210
Smoking	1.4	1.1–1.9	0.010
cTnI (ng/mL)	2.0	1.5–2.8	<0.001
LVEF (%)	1.72	1.45–2.25	0.005
eGFR	0.99	0.98–1.00	0.065
Triglyceride/HDL Ratio (THR)	1.73	1.40–2.30	0.020
Triglyceride–Glucose Index (TGI)	1.82	1.50–2.80	0.002
Pan-Immune-Inflammation Value (PIV)	1.91	1.61–2.70	0.003

**Table 4:** ROC analysis results in patients with STEMI.

Parameters	AUC	95% (CI)	Sensitivity	Specificity	Cut-Off	*p* Value
Age (years)	0.65	0.60–0.70	75%	55%	65<	0.042
Total Cholesterol (mg/dL)	0.66	0.61–0.71	72%	60%	200<	0.030
Triglycerides (mg/dL)	0.64	0.59–0.69	70%	58%	150<	0.040
Diabetes Mellitus	0.68	0.63–0.73	75%	60%	-	0.041
Smoking	0.70	0.65–0.75	78%	62%	-	0.010
cTnI (ng/mL)	0.85	0.80–0.90	90%	75%	0.1<	<0.001
LVEF (%)	0.75	0.70–0.80	82%	65%	50%>	0.005
Triglyceride/HDL Ratio (THR)	0.67	0.62–0.72	73%	60%	3.5<	0.020
Triglyceride–Glucose Index (TGI)	0.70	0.65–0.75	78%	64%	8.5<	0.015
Pan-Immune-Inflammation Value (PIV)	0.72	0.67–0.77	80%	66%	350<	0.012
Combination of TGI, THR, and PIV	0.78	0.73–0.83	83%	70%	-	<0.001
Combination of TGI, THR, PIV, cTnI	0.88	0.84–0.92	85%	75%	-	<0.001

**Table 5:** Multiple logistic regression analysis of factors used for mortality in patients with ACS.

Parameters	Odds Ratio (OR)	95% CI	*p* Value
Age (years)	1.04	1.02–1.06	0.001
Total Cholesterol (mg/dL)	1.02	1.01–1.03	0.002
Triglycerides (mg/dL)	1.01	1.00–1.02	0.010
Diabetes Mellitus	1.5	1.2–1.9	0.003
Smoking	1.7	1.3–2.2	0.001
cTnI (ng/mL)	2.4	1.8–3.2	<0.001
LVEF (%)	1.4	1.1–1.87	0.002
Triglyceride/HDL Ratio (THR)	2.0	1.5–2.6	0.015
Triglyceride–Glucose Index (TGI)	2.2	1.7–2.9	0.001
Pan-Immune-Inflammation Value (PIV)	2.5	1.9–3.3	<0.001

**Table 6:** ROC analysis results in patients with mortality in patients with ACS.

Parameters	AUC	95% (CI)	Sensitivity	Specificity	Cut-Off	*p* Value
Age (years)	0.70	0.65–0.75	75%	60%	65<	0.001
Total Cholesterol (mg/dL)	0.68	0.63–0.73	70%	58%	200<	0.005
Triglycerides (mg/dL)	0.66	0.61–0.71	72%	55%	150<	0.010
Diabetes Mellitus	0.75	0.70–0.80	78%	62%	-	0.002
Smoking	0.72	0.67–0.77	76%	60%	-	0.003
cTnI (ng/mL)	0.85	0.80–0.90	85%	70%	0.1<	<0.001
LVEF (%)	0.78	0.73–0.83	80%	65%	50%>	0.002
Triglyceride/HDL Ratio (THR)	0.75	0.70–0.80	78%	63%	3.5<	0.004
Triglyceride–Glucose Index (TGI)	0.78	0.73–0.83	80%	68%	8.5<	0.007
Pan-Immune-Inflammation Value (PIV)	0.80	0.75–0.85	82%	67%	370<	0.009
Combination of TGI, THR, and PIV	0.85	0.81–0.89	86%	74%	-	<0.001
Combination of TGI, THR, PIV, cTnI	0.92	0.88–0.96	89%	80%	-	<0.001

## Data Availability

The original contributions presented in the study are included in the article, and further inquiries can be directed to the corresponding author.
